# Hand hygiene compliance in intensive care units: An observational study

**DOI:** 10.1111/ijn.12789

**Published:** 2019-10-31

**Authors:** Magdalena Hoffmann, Gerald Sendlhofer, Veronika Gombotz, Gudrun Pregartner, Renate Zierler, Christine Schwarz, Christa Tax, Gernot Brunner

**Affiliations:** ^1^ Executive Department for Quality and Risk Management University Hospital Graz Graz Austria; ^2^ Division of Endocrinology and Diabetology, Department of Internal Medicine Medical University of Graz Graz Austria; ^3^ Research Unit for Safety in Health, Division of Plastic, Aesthetic and Reconstructive Surgery, Department of Surgery Medical University of Graz Graz Austria; ^4^ Institute for Medical Informatics, Statistics and Documentation Medical University of Graz Graz Austria; ^5^ Department of Surgery University Hospital Graz Graz Austria; ^6^ University Hospital Graz Graz Austria

**Keywords:** compliance, culture, hand disinfection, hand hygiene, intensive care, nursing

## Abstract

**Aim:**

Health care–associated infections along with antibiotic resistance are a leading risk for patient safety in intensive care units. Hygienic hand disinfection is still regarded as the most effective, simplest, and most cost‐effective measure to reduce health care–associated infections. To improve hand hygiene compliance and to prevent health care–associated infections, interventions of the “German Clean Hands Campaign” were implemented in a university hospital.

**Methods:**

Observational single‐center study using direct observation and feedback. Hand hygiene performance was assessed in 12 intensive care units between 2013 and 2017. Linear mixed model regression analyses were used to estimate the compliance trend over time.

**Results:**

In total, 10 315 “my five moments for hand hygiene” were observed. The mean hand hygiene compliance rates increased from 75.1% to 88.6% during the study period, yielding an estimated increase of about 4.5% per year. However, there are differences in compliance between occupational groups (physicians: between 61.2% and 77.1%; nurses: between 80.2% and 90.9%; others: between 61.3% and 82.4%).

**Conclusions:**

After implementation of the “German Clean Hands Campaign” interventions, an overall significant improvement of hand hygiene was detected. Compliance measurements helped to raise awareness among health care professional groups.

## SUMMARY STATEMENT

What is already known about this topic?

- A significantly higher prevalence of infections has been observed in intensive care unit patients compared with patients in other wards.
- Hygienic hand disinfection is still regarded as the most effective, simplest, and most cost‐effective measure to reduce health care–associated infections.
- Low hand hygiene rates in intensive care units are a major problem for patient safety.

What is already known about this topic?

- Implementation of the “German Clean Hands Campaign” showed continuous improvement in hand hygiene for all health care professionals in intensive care units over a period of 5 years.
- There are differences in compliance rates between health care profession and intensive care unit types.
- Pediatric intensive care units had the highest hand hygiene compliance rates.

The implications of this paper:

- Overall, hand hygiene compliance in intensive care units increased over a period of 5 years after continuous campaigning, training, observation, compliance measurements, and direct feedback.
- It is necessary to raise awareness for hand hygiene in a repetitive manner within all health care professional groups now and in the future.
- Because of the differences in compliance rates between health care professionals, more tailored and evidence‐based interventions should be implemented.

## 1 INTRODUCTION

Health care–associated infections (HAIs), along with antibiotic resistance, are one of the leading risks for patient safety in intensive care units (ICUs) and cause a serious disease burden including economic impact (Sendlhofer et al., 2015). HAIs have become a growing threat to global public health with more than 2.6 million cases of HAIs in the European Union and European Economic Area each year (Cassini et al., 2016). Marchetti et al. reported in 2013 that HAIs in acute‐care hospitals of the United States of America lead to direct and indirect costs, totaling $96 to $147 billion annually (Marchetti & Rossiter, 2013). The enormous clinical and economic burden of infection places HAIs high on the list of devastating and costly illnesses, such as cancer, heart attack, stroke, and diabetes, thereby mandating further research and greater efforts to contain a pressing health care.

The Word Health Organization (WHO), a long‐standing leading authority in campaigning hand hygiene (HH), urges every country to strengthen infection prevention and control, and appeals for networking with stakeholders to take better action for the prevention of HAIs (Saito, Kilpatrick, & Pittet, 2018). HAIs are still a substantial burden among infectious diseases, exceeding the burden of other infections such as influenza and tuberculosis (Cassini et al., 2016). A significantly higher prevalence of infections such as pneumonia and infections of the lower respiratory tract, bloodstream, and surgical site, has been observed in ICU patients compared with patients of other wards (Cairns, Reilly, & Booth, 2010). A more recent published study in 2017 showed a 39% prevalence of HAIs in Polish adult ICU patients (Deptuła et al., 2017).

Hygienic hand disinfection is still regarded as the most effective, simplest, and most cost‐effective measure to reduce HAIs (Hugonnet, Perneger, & Pittet, 2002a; Kampf, Löffler, & Gastmeier, 2009; Sickbert‐Bennett et al., 2016; Thomas von Lengerke et al., 2017). However, low HH rates in ICUs are a major problem (Erasmus et al., 2010; Musu et al., 2017). In 2017, it was reported that around half of the ICUs from six hospitals in the North of Italy are lacking standard operation procedures (SOP) for behavioral hygiene. Even where SOPs were available, they were not implemented in daily routine (Musu et al., 2017). A Belgian study published in 2015 showed that after 10 years of campaigning HH with the application of different interventions, only compliance rates of 80% were achieved (Fonguh, Uwineza, Catry, & Simon, 2016). Compliance rates from Saudi Arabia showed that in 41% of cases, no HH compliance interventions were carried out at all by health care professionals (Mahfouz, Gamal, & Al‐Azraqi, 2013). Large differences for HH compliance rates between health care professional groups have been demonstrated in pediatric ICUs. However, they are often higher than in adult ICUs (Mahfouz et al., 2013).

In 2005, the WHO released the global campaign “Clean Care is Safer Care” (World Health Organization, 2009) to promote HH for infection prevention and patient safety. Out of this initiative, the “German Clean Hands Campaign” (GCHC) was established in January 2008 (Reichardt et al., 2013) to support the implementation of interventions and to prevent HAIs in health care institutions and hospitals.

Here, we implemented the interventions of the GCHC in an iterative process of information, training, and direct feedback. Hand disinfection was monitored as “my five moments” (M5M) for HH in the following situations: #1 before patient contact, #2 before aseptic tasks, #3 after exposure to bodily fluids, #4 after patients contact, and #5 after contact with patients' surroundings. The overall aim of this study was to improve HH compliance and to prevent health care–associated infections by implementing interventions of the “German Clean Hands Campaign” in a university hospital, to evaluate differences in staff groups, types of ICU, and parts of M5M.

## 2 METHODS

### 2.1 Study design

To determine the effectiveness of the implementation of GCHC, an observational single‐center study was performed between 2013 and 2017 to assess compliance rates of different health care professionals (physicians, nurses, and others: health care professionals such as medical technical assistants, dieticians, physiotherapists, occupational therapist) in different ICU types and for each of M5M indications for HH.

### 2.2 Participants and setting

Twelve ICUs at an Austrian University Hospital, six surgical ICUs with a total of 68 beds, two medical ICUs with 34 beds, and four pediatric ICUs with 50 beds.

### 2.3 Implemented interventions of GCHC

Starting in 2012, several interventions of GCHC were implemented in an iterative process in all ICUs of a university hospital. These included training of 506 health care professionals over a 5‐year period by hygiene experts through theoretical lectures and practical demonstrations. Furthermore, comprehensive equipment with disinfectant dispensers were installed for each ICU bed, and video clips on HH training as well as new or revised SOPs on HH were provided. In total, 62 HH conferences and clinical events—the so‐called “Day of Action for HH”**—**were organized. Moreover, an in‐house HH newsletter was delivered quarterly to all health care professionals. A more detailed description of the implemented interventions is outlined in a previous publication (Hoffmann et al., 2018).

### 2.4 Compliance measurements

To evaluate the HH compliance of health care professionals in the participating ICUs, four direct announced observations by two hygiene experts (nurses with additional training in hygiene and professional experience of at least 1 year) were performed between 2013 and 2017 in each ICU, approximately spaced 1 year apart. Observations were carried out during daily routine patient care, strictly following the guideline for conducting compliance measurements issued by the GCHC (https://www.aktion‐sauberehaende.de/ash/messmethoden/). Each indication of M5M for HH was observed during day shifts (different moments) for at least 20 times during one compliance measurement on one observational day. A minimum of at least 150 M5M of HH was observed in each ICU in total.

All observed M5M for HH were documented in the online tool “webkess” (https://webkess2/webkess2/de-DE//Home/Index),%20 which is connected with the “National Reference Centres for Surveillance” (NRZ), Berlin, Germany (www.nrz‐hygiene.de).

After each compliance measurement, the hygiene experts provided direct feedback about the performance to all observed health care professionals. Thereafter, a report was also compiled and sent out to the heads of the ICUs as well as to the hospital management. If the reported compliance rate within a unit was below 80%, the hygiene experts issued and conducted an appropriate follow‐up training.

### 2.5 Ethical considerations

The study was approved by the Ethics Committee of the Medical University of Graz (vote#: 29‐458 ex 16/17). Each ward was informed by their managers in advance that an observation would be performed in the near future. There were no patients involved in the study, so consent was not applicable. The research and reporting methodology followed SQUIRE 2.0.

### 2.6 Data analysis

The compliance data were descriptively summarized using means and standard deviations at each of the four measurement times as well as dot plots for visualization. Linear mixed model regression analyses were used to estimate an overall compliance trend over the years of observation as well as health care profession‐specific and M5M‐specific trends. Because of repeated measurements, the different wards were used as random effects. Results are presented along with their 95% confidence intervals (CI) and are depicted using trend charts. All analyses were performed using *R* version 3.4.4.

## 3 RESULTS

In sum, 12 ICUs with their health care professionals participated in this observational study. In total, 48 compliance measurements with 10 315 (physicians n = 1.417, nurses n = 7.898, others n = 1.000) observed M5M for HH were conducted in the observational period.

**Figure 1:**
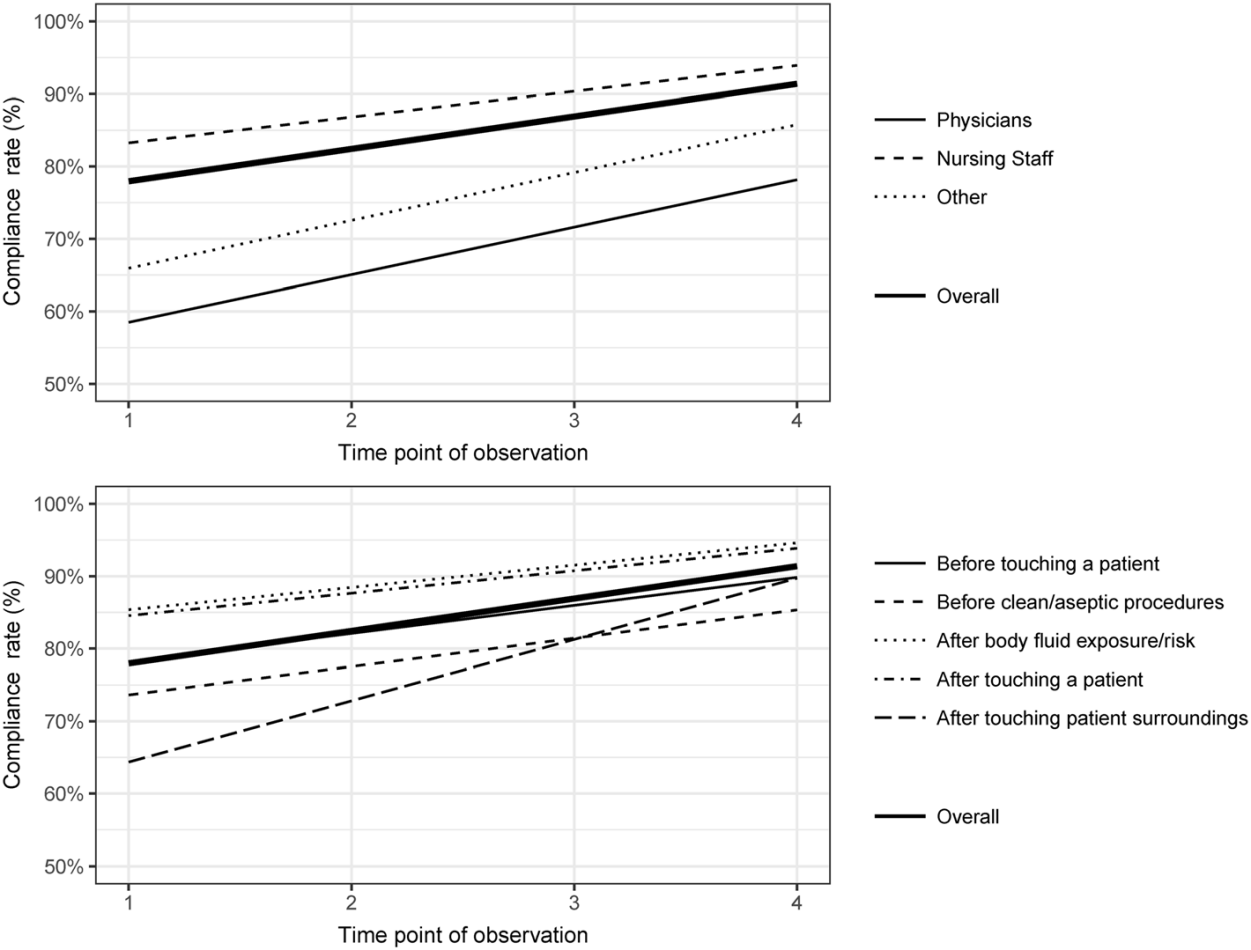
Trend plot depicting the estimated overall trend as well as health care profession and M5M specific trends

Per year, the overall mean (±standard deviation) HH compliance rates for all ICUs was 75.1% (12.3), 85.4% (7.4), 89.6% (6.6), and 88.6% (8.1). The estimated increase per year was 4.5% (95% CI, 2.7‐6.3%; *P* < .001). The overall trend is presented in Figure 1 and values are presented in Table S1.

We observed differences between the ICU types. Mean compliance rates in surgical ICUs (n = 6) increased from 68.8% (3.7) to 82.2% (4.2) and that of medical ICUs (n = 2) from 69.1% (22.6) to 89.5% (6.9). Pediatric ICUs (n = 4) already started considerably higher at 87.6% (7.2) and were able to improve further to 97.8% (1.5).

**Figure 2:**
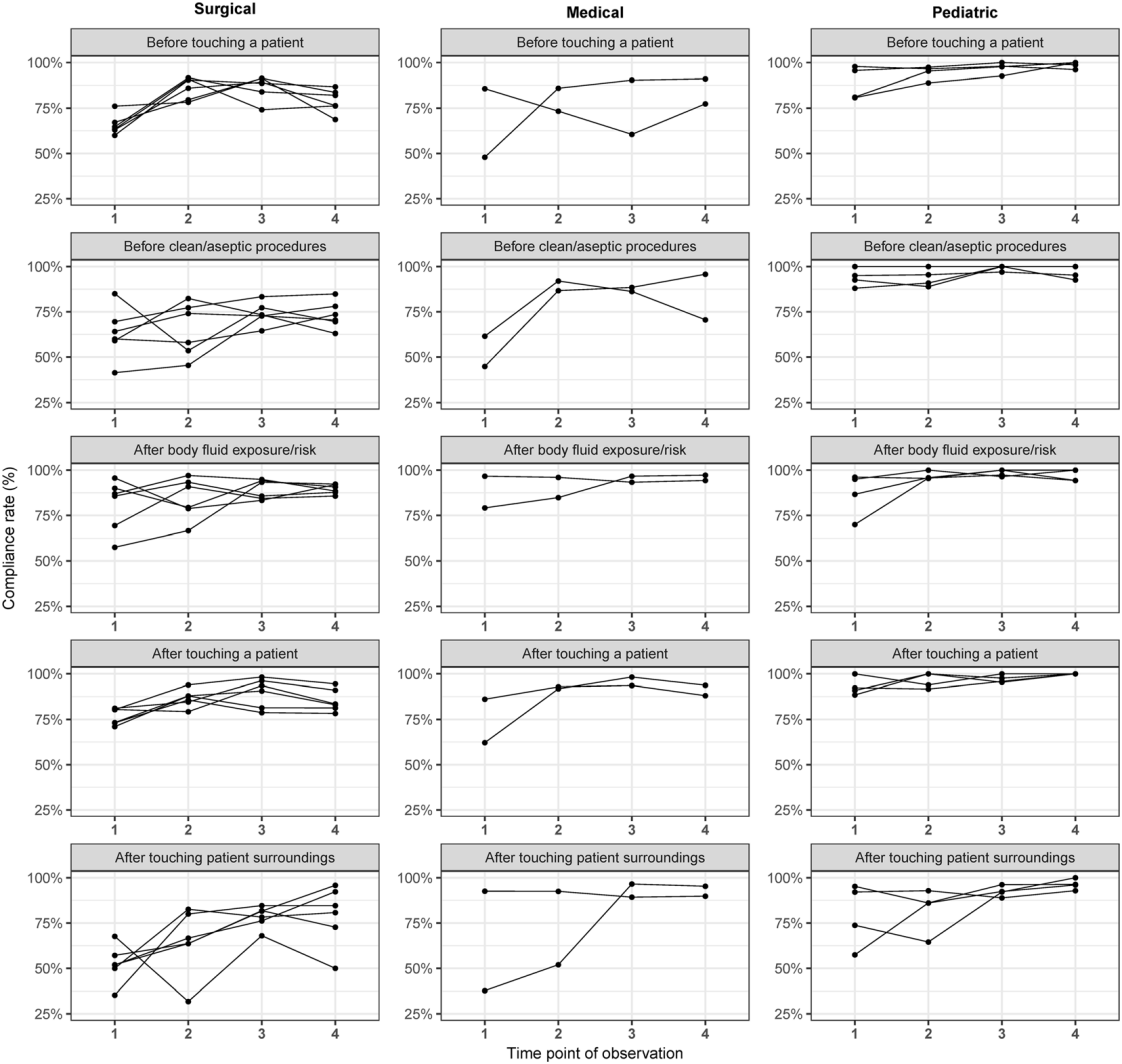
Compliance rates by indication of my five moments (M5M) and intensive care unit (ICU) type. Lines represent the compliance rates of each ICU type

**Figure 3:**
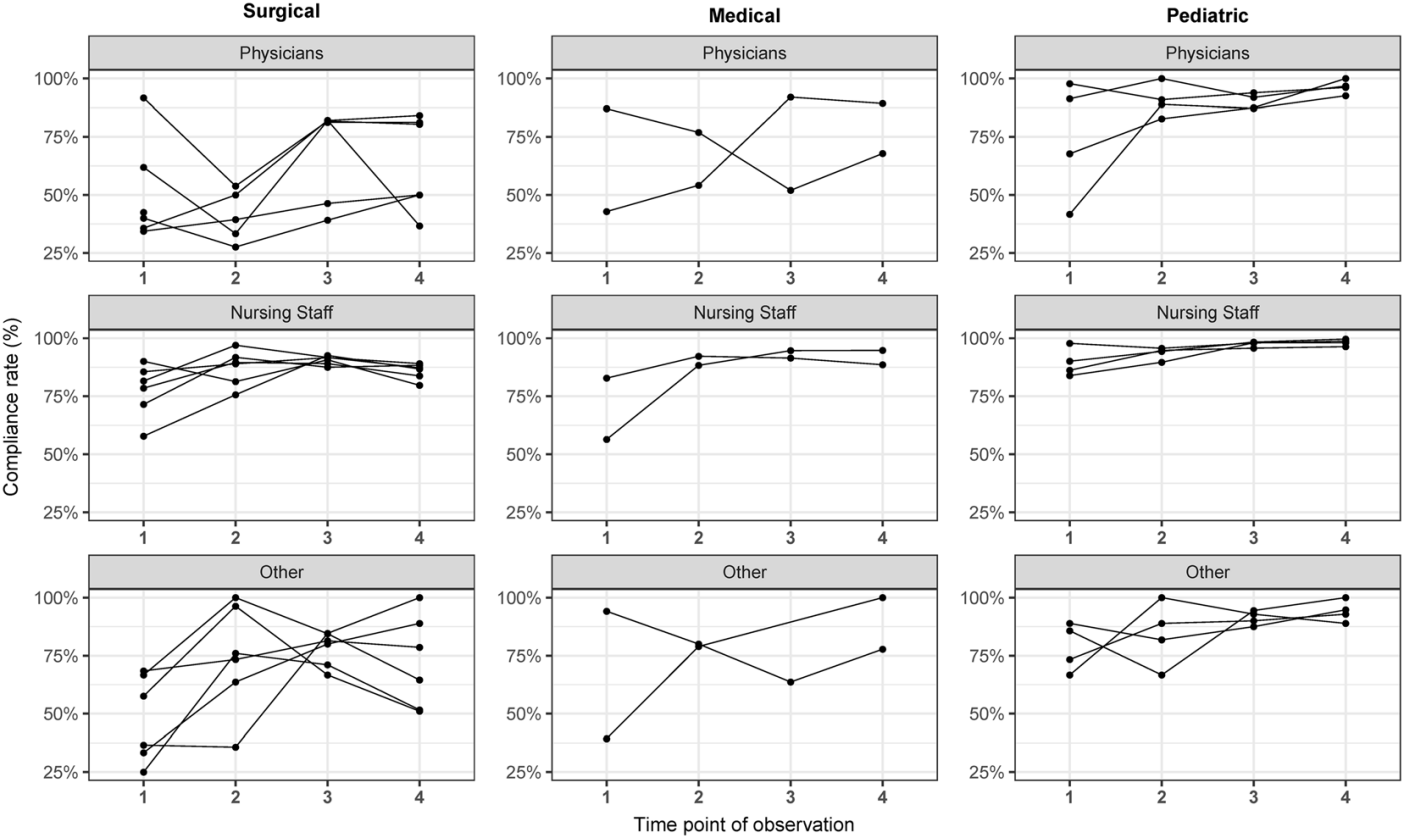
Compliance rates by health care profession and intensive care unit (ICU) type. Lines represent the compliance rates of each ICU

Furthermore, compliance rates were analyzed separately (Figure 2) for each of the five M5M for HH (#1 before patient contact, #2 before aseptic tasks, #3 after exposure to bodily fluids, #4 after patients contact, and #5 after contact with patients' surroundings). Individual compliance rates for each health care profession (physicians, nurses, others) and for each type (surgical, medical, and pediatric) are presented in Figure 3.

### 3.1 Compliance rates for the M5M for HH

Overall mean compliance rates for #1 increased from 73.6% (15.1) to 86.4% (10.7). For #2, mean compliance rates increased from 71.8% (19.8) to 82.8% (13.4). For #3, compliance rates increased from 84.1% (12.6) to 93.1% (4.6). For #4, mean compliance rates increased from 81.6% (10.6) to 91.1% (8.1). Finally, for #5, compliance rates increased from 63.6% (20.8) to 87.2% (14.1). The trend estimates for each of the M5M are shown in Figure 1 **.**

### 3.2 C**ompliance rates by health care profession**

The mean compliance rate for physicians was 61.2% (24.8) and increased to 77.1% (21.2). Mean compliance for nurses was 80.2% (12.6) and increased to 90.9% (6.4). Finally, the mean compliance rate for other professions was 61.3% (23.2) and increased to 82.4% (18.0). The trend estimates for each of the health care professions are presented in Figure 1.

## 4 DISCUSSION

The aim of this study was to assess the HH compliance rate of health care professionals in all ICUs in a university hospital over a 5‐year period. Results showed an overall increase of the compliance rates, however, there were noticeable differences in compliance rates of the M5M for HH indication, profession, and ICU type. Furthermore, there were fluctuations over the individual years after the implementation of GCHC. It seems that interventions differently effected compliance throughout indications, professions, and ICU types.

Comparing these data to reference data of the GCHC with more than 700 participating hospitals in the German‐speaking area, we were able to demonstrate higher compliance rates for ICUs than the stated average (http://www.aktion‐sauberehaende.de). In general, compliance rates in our ICUs were very high compared with others (Erasmus et al., 2010; Stahmeyer, Lutze, Lengerke, Chaberny, & Krauth, 2017).

Regarding different health care professions, nurses had the highest compliance rate, which is a well‐known phenomenon in the health care setting (Sharma, Sharma, & Koushal, 2012). Furthermore, our findings were comparable with further studies, where similar results for physicians in ICUs were achieved (Hugonnet, Perneger, & Pittet, 2002a; Laskar et al., 2018; Pittet et al., 2004).

When comparing different ICU types, pediatric ICUs often have the highest HH compliance rates. These differences might be because of a general careful attitude of health care professionals working with pediatric patients and with their close relatives during the hospital stay. However, the sample size in the subgroups were small, therefore, results should be interpreted accordingly.

Looking at the results for the individual ICUs, there are increasing and decreasing HH compliance rates over the years. Most of the observed compliance rates increased after the initial compliance measurement and decreased slightly thereafter. A systematic review performed in 2016 showed that adopting a multimodal approach to improve HH, whether guided by the WHO framework or by another tested multimodal framework, resulted only in moderate improvements in HH compliance over time (Kingston, O'Connell, & Dunne, 2016). The question thus arises how long measures have a positive impact on compliance rates, and when and why they are likely to decrease again. In order to prevent a decrease, it seems to be necessary to raise awareness of HH in a repetitive manner within all health care professional groups. It should be considered that the motivation for improving HH should derive from each health care professional and not only from hygiene experts (T. von Lengerke et al., 2015).

So far, it is also unclear whether accompanying interventions such as multidisciplinary SOPs instead of monodisciplinary SOPs are needed in order to raise awareness. So far, one study demonstrated that SOPs that were created by a multiprofessional team in order to improve the clinical workflow at the ICU also helped to improve compliance rates (Scheithauer & Lemmen, 2013). Education of health care professionals, written and verbal reminders, different types of performance feedback, administrative support and staff involvement, adequate staffing, workload control, engagement of champions, and positive organizational culture could also improve low compliance rates in HH and thus increase patient safety (Neo, Sagha‐Zadeh, Vielemeyer, & Franklin, 2016; Zingg et al., 2014). However, a study of von Lengerke et al., 2017 revealed that individual interventions were not equally effective for each health care professional group (Thomas von Lengerke et al., 2017). This is also the conclusion of a Cochrane review of 2017. Therefore, with the identified variability in certainty of evidence, interventions, and methods, there remains an urgent need to undertake methodologically robust research to explore the effectiveness of multimodal versus simple interventions to increase HH compliance. Further, it is important to identify which components of multimodal interventions or combinations of strategies are most effective in a particular context (Gould, Moralejo, Drey, Chudleigh, & Taljaard, 2017).

HH is not just an important topic for health care professionals. In a previous study, we also found that relatives of ICU patients ranked germs and hygiene at ICUs as the second most important information topic (Hoffmann et al., 2018). This raises the question if a greater involvement of patients and relatives might also change behaviors towards HH. This might also explain why higher compliance rates were observed in pediatric ICUs, where relatives (eg, parents or legal guardians) were directly involved in the care of their children as well as in HH. A recently published study by Zhao, Yang, Huang, and Chen (2018) on making HH interventions more attractive to nurses showed that the most attractive intervention for nurses was to provide solid evidence of HH and its effectiveness in infection control (Zhao et al., 2018). Therefore, future interventions should be more tailored to the specific needs of each health care professional group.

Nevertheless, there are also often certain barriers for HH. For example, a study in emergency departments revealed ambiguity about when to clean one's hands, the pace and urgency of work, environmental/operational issues, or sore hands as such barriers (Jeanes, Coen, Drey, & Gould, 2018). It is most likely that these barriers also exist in ICUs. Further research should therefore focus on the elimination of such barriers.

### 4.1 Strengths and limitations

The biggest strength of this study is that HH compliance rates were observed consistently in same ICU types over several years, with a high amount of observed indications for HH. Furthermore, a large proportion of health care professionals as part of our information and training program were informed, trained, and finally directly involved during HH compliance measurements. A major limitation was the diversity of the implemented interventions. No conclusions can be drawn on the effectiveness of an individual measure. Another limitation was that compliance measurements were performed by internal hygiene experts (A. Jeanes, Coen, Wilson, Drey, & Gould, 2015). Furthermore, the so‐called “Hawthorne Effect” questions the method of compliance measurements and their results in general (Hagel et al., 2015; McLaws & Kwok, 2018). Finally, measured HH compliance rates were not compared with infection control tools according to international standards (Zingg et al., 2014).

## 5 CONCLUSION

The study showed an overall increase of HH compliance rates after the implementation of GCHC interventions. The results are encouraging, but raise the question of whether more interventions and which in particular help to raise HH compliance rates. It is necessary to increase awareness for HH in a repetitive manner now and in the future. Because of the differences in compliance rates between health care professionals, more tailored and evidence‐based interventions should be implemented. Further, data are also needed on the education and empowerment of patients and their relatives, which also seem to have an effect on HH and hence compliance rates.

## FUNDING SOURCES

There was no funding.

## CONSENT FOR PUBLICATION

Not applicable.

## AVAILABILITY OF DATA AND MATERIAL

The datasets used and/or analyzed during the current study are available from the corresponding author on reasonable request.

## CONFLICT OF INTEREST

The authors have declared that no competing interests exist.

## SUBMISSION DECLARATION

This present work has not been published previously, is not under consideration for publication elsewhere, and will not be published elsewhere in the same form. All authors approved publication of this article.

## AUTHORSHIP STATEMENT

MH wrote the manuscript. GS, VG, and RZ, designed and performed the study; MH, CT, GS, CS, GB interpreted data and contributed to discussions; GP performed statistical analysis; and GB and GS supervised the project.

## Supporting information


**Table S1:** Estimated trend (compliance increase per observation) for each indication of M5M and healthcare profession
