# Supplementary material for: Hand hygiene compliance in intensive care units: An observational study
Source: Int J Nurs Pract. 2019 Oct 31;26(2):e12789. doi: 10.1111/ijn.12789 (PMC9285823; doi:10.1111/ijn.12789)
Supplement: Supplementary file 1 — Table S1: Estimated trend (compliance increase per observation) for each indication of M5M and healthcare profession [file IJN-26-0-s001.docx]

Supplemental Material

Table 1: Estimated trend (compliance increase per observation) for each M5M and healthcare profession

|  | Estimate | 95% CI | p-value |
| --- | --- | --- | --- |
| Before touching a patient | 3.88 | 1.27-6.49 | 0.005 |
| Before clean/aseptic procedures | 3.90 | 1.20-6.60 | 0.006 |
| After body fluid exposure/risk | 3.08 | 1.12-5.04 | 0.003 |
| After touching a patient | 3.11 | 1.48-4.73 | <0.001 |
| After touching patient surroundings | 8.46 | 5.03-11.88 | <0.001 |
| Physicians | 6.55 | 2.00-11.10 | 0.006 |
| Nursing staff | 3.55 | 1.65-5.46 | 0.001 |
| Other | 6.62 | 2.09-11.15 | 0.005 |
